# An Agro-Climatic Approach to Developing a National Prevention Tool for Deoxynivalenol in French Maize-Growing Areas

**DOI:** 10.3390/toxins14020074

**Published:** 2022-01-20

**Authors:** Agathe Roucou, Christophe Bergez, Benoît Méléard, Béatrice Orlando

**Affiliations:** Arvalis-Institut du Végétal, Station Expérimentale, 91720 Boigneville, France; christophe.bergez@free.fr (C.B.); b.meleard@arvalis.fr (B.M.); b.orlando@arvalis.fr (B.O.)

**Keywords:** maize, *Fusarium graminearum*, deoxynivalenol, climatic conditions, residue management, agricultural practices, tool management

## Abstract

The levels of deoxynivalenol (DON)—a mycotoxin produced by *Fusarium graminearum—*in maize for food and feed are subject to European Union regulations. Obtaining a compliant harvest requires the identification of agronomic and climatic risk factors related to higher fungal contamination and DON production. A national, multiyear database for maize was created, based on field survey data collected from 2004 to 2020. This database contains information about agricultural practices, climatic sequences and DON content at harvest for a total of 2032 maize fields localized in the French maize-growing regions. A linear mixed-model approach highlighted the presence of borers, late harvest and inadequate crop residue management, normal-to-cold temperatures in March, humidity in August and the absence of a hot end of the maize development cycle with a dry August as creating conditions favoring maize contamination with DON. The various possible associations between these risky climatic conditions and agricultural practices were compared, grouped and ranked as related to very low to high DON concentrations. Some combinations may even exceed the regulatory threshold. The national prevention tool, created for producers and agricultural cooperatives, is informative and easy-to-use to control the sanitary quality of their harvest.

## 1. Introduction

France is the second largest producer of maize in Europe, with a harvest of 13 million tonnes from an area of 1.5 million hectares in 2020 [1]. Mycotoxins, produced by fungi, can commonly contaminate maize, before harvest, but also during grain storage. Deoxynivalenol (DON) is the most widespread trichothecene mycotoxin, a group of mycotoxins frequently found in food and feed, and is one of the most important mycotoxins affecting the sanitary quality of maize in the European Union (EU) [2]. In France, DON is produced principally by *Fusarium graminearum,* from the genus *Gibberella*. DON, also known as vomitoxin, may have strong emetic effects if consumed in high quantities [3], and it also decreases grain yield [4]. Its presence in maize is, therefore, a major health and safety concern [3,5]. Similar to many other countries worldwide, the EU has imposed regulations for the levels of DON permissible in food, based on the health risk associated with DON consumption (Commission Regulation 1126/2007). The maximum permissible level of DON in unprocessed maize intended for human consumption is currently 1750 µg/kg. As regards feed, Commission Recommendation 2006/576/EC on the presence mycotoxins in products intended for animal feed recommends a guidance value of 8000 µg/kg for cereals, including maize. Advances in knowledge and technology have enabled producers and processors to improve maize production, treatment and storage methods, to decrease the likelihood of mycotoxins exceeding regulatory thresholds in their produce [6,7,8,9]. Plant breeding has been touted as the safest and most effective way to reduce DON contamination in maize crops [10,11,12]. Until now, no maize genotypes highly resistant to infection with *F. graminearum* or to DON contamination have been produced yet [11]. Moreover, France has banned the cultivation of genetically modified organisms (GMOs) on its territory [13]. In this context, there is a need for more effective management strategies to prevent contamination with this mycotoxin. In particular, preharvest strategies could be used to prevent, or at least limit, DON contamination in the field [14]. Such approaches require the identification of risk factors influencing contamination and the production of DON by *F. graminearum* in the field, which can then be targeted by maize producers and processors.

*Fusarium graminearum* infection and growth, and DON production, are dependent on favorable agronomic conditions. These include kernel damage due to borer insects, related to higher DON accumulation [15,16]. The wounds created by insects provide a route of entry for the fungus, distributing fungal propagules as they feed and proliferate, and for microconidia or mycelia already present on the ear tissues [17]. In North America and Europe, kernel infection, closely correlated with insect injury, appears to be a major infection pathway [18,19,20]. Harvest date has also been highlighted as a risk factor for DON contamination. Experiments in Europe and in New Zealand have shown that the risk of DON contamination tends to increase with later harvest dates [21,22,23]. This may reflect the longer time period available for *F. graminearum* to grow and produce toxins [23]. A late harvest may also shift the susceptible stages to more favorable climatic periods for *F. graminearum* growth, such as late rains, which provide moisture, favoring fungal development [24]. The presence of large amounts of residues from previous crops, such as maize stalks and grains, is considered to constitute a major source of inoculum for *F. graminearum* [25]. The fungus can survive as a saprophyte on the residues of previous crops for two or more years [26]. In this context, leaving unaltered infected residues on the surface can increase fungal survival. Any practices resulting in the removal, destruction or burial of infected residues, such as crushing and ploughing, is likely to reduce the amount of inoculum [23,27,28,29]. The management of these residues, mostly through soil tillage (ploughing), is therefore a key element of the cropping practices for decreasing the density of infected residues on the soil surface, making it possible to decrease (1) inoculum production, (2) the number of spores available for dispersal and (3) dispersal itself [30]. These three agronomic practices (removal, destruction and burial of infected residues) create unfavorable conditions for the growth and development of *F. graminearum*, and for DON production. A better understanding of their particular and cumulative impacts would make it possible to adapt agricultural management in the field [22,30]. In addition, climatic factors can create favorable conditions for fungal growth and mycotoxin production at particular times in the plant development cycle. It is, therefore, necessary to identify high-risk climatic sequences to adapt the preventive strategy in the field.

*Fusarium graminearum* infection and growth and DON production result from the complex interaction of several climatic factors at key periods of the maize development cycle. *F. graminearum* first forms perithecia (fruiting bodies) on the residue. These structures can develop over a wide range of temperatures (5–30 °C) [31]. They then forcibly discharge ascospores into the air [10], this process having an optimal temperature of 16 °C [32,33]. Finally, the sporulation, germination and growth of *F. graminearum* are optimal at 24–26 °C [10,34], whereas DON production is optimal between 28 and 30 °C [35]. *F. graminearum* infects maize kernels principally via the silks, which makes flowering a sensitive period for plant infection [36]. Wounds on the plant, such as those created by borers, can act as points of entry for the fungus [20], leading to contamination during phenological stages other than flowering. Climatic conditions during flowering, including humidity levels in particular, therefore play an important role in maize infection [31,37]. In Serbia, following a period of extremely rainy weather, almost 50% of the samples analyzed were contaminated with DON at concentrations exceeding 1750 µg/kg [38]. Indeed, rain plays an important role in the dispersal of the fungus: spores are splash-dispersed during rainfall events, enabling them to reach plant spikes and spread over the canopy [39]. DON production follows the same trend as fungal growth, increasing during a cool and wet growing season [40], however, its dependence on weather conditions during the final maize ripening period is different, particularly as concerns the occurrence of rain [41]. Ripening is, therefore, also a very sensitive period for contamination and fungal development. Combination of favorable agronomic and climatic conditions can create a prosperous environment for both fungal contamination and DON production during the sensitive periods of the plant. In the field, risky situations must be highlighted during maize development. Indeed, their early identification makes it possible to develop appropriate preventive strategies before harvesting.

DON may further accumulate during the post-harvest period, mostly during grain transportation and storage [6], but the implementation of good practices involving drying and storage management has been shown to prevent the further production and accumulation of DON after harvest [9]. Preharvest management in the field can reduce the amount of contaminated maize, by separating contaminated batches from clean batches at harvest. The use of a preventive strategy in the field can facilitate this approach, through targeting of the maize fields most likely to have high levels of DON concentrations. The aim of this work was, therefore, to identify easy-to-target combinations of agronomic and climatic risk factors promoting high DON content in maize, possibly exceeding regulatory thresholds, which could then be used in the development of preharvest management tools for use in the field.

## 2. Results

### 2.1. Association of Agronomic Factors Influencing DON Concentrations

An analysis of variance (ANOVA) was performed on DON concentrations, at the national scale, in France. The presence of borers and the categories of sowing and harvest dates were the only agronomic factors found to have independent significant effects (*p*-value < 0.001, for the three).

**Figure 1:**
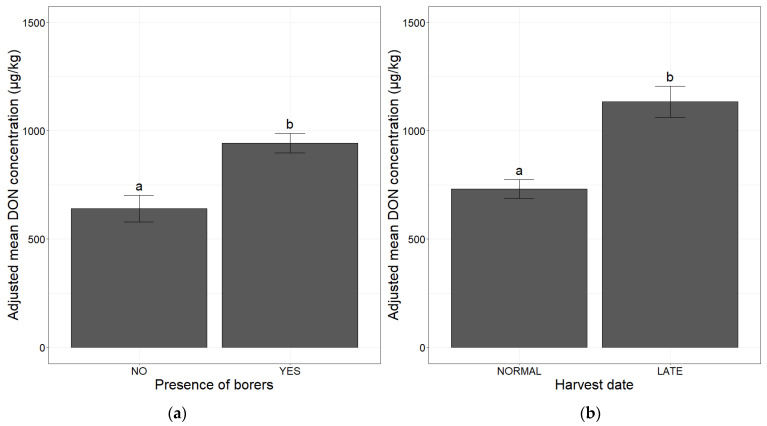
Mean deoxynivalenol (DON) concentration adjusted for agronomic factors, in maize. (**a**) Mean adjusted DON concentration in relation to the presence or absence of borers; (**b**) mean adjusted DON concentration according to harvest date. The two categories for the presence of borers (NO, YES) and for harvest date (NORMAL, LATE) are shown on the *x*-axis. The adjusted mean DON concentration obtained with a mixed linear model (lmer(DON ~ factor + (1|Year))) applied to a database of 2032 observations is plotted on the *y*-axis. Different letters (a and b) above the bars indicate significant differences (*p*-value <0.001) in ANOVA for each of the variables.

The presence of borers in agricultural plots significantly increased the adjusted mean DON concentration (value multiplied by 1.5 relative to plots without borers) (Figure 1a). Late harvesting significantly increased susceptibility to DON contamination (multiplied by 1.6) relative to normal harvesting dates (Figure 1b).

Inadequate residue management appeared to increase the risk of DON contamination (value multiplied by 1.18 compared to plots with adequate residue management), but this effect was not significant (*p*-value *>* 0.05, ANOVA).

**Figure 2:**
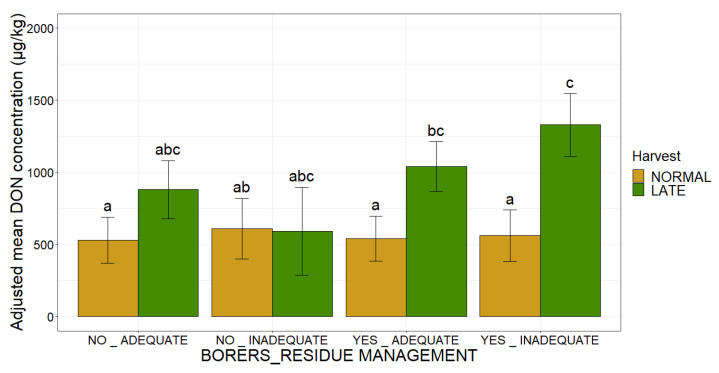
Adjusted mean DON concentrations, according to the presence of borers, residue management and harvest date. The presence of borers and the residue management are plotted along the *x*-axis (NO_ADEQUATE, NO_INADEQUATE, YES_ADEQUATE, YES_INADEQUATE) with bars of two colors corresponding to the two harvesting dates. Green bars correspond to plots with late harvest dates, whereas yellow bars correspond to plots with normal harvest dates. The adjusted mean DON concentration obtained by applying the mixed linear model (lmer(DON ~ Borers × Residue_management × Harvest + (1|Year))) to a database of 2032 observations is plotted on the *y*-axis. Different letters (a, b and c) above the bars indicate significant differences (*p*-value < 0.05) in Tukey’s multiple comparison test.

The combination for the three agronomic factors had a significant impact on DON contamination (R^2^ = 0.02, *p*-value < 0.001). Adjusted mean DON concentration increased as soon as one of the three factors was in the “at risk” category, with the largest increases for an individual factor observed for harvest date. The largest increase was observed when all three factors were in the “at risk” category (Figure 2). Thus, plots infested with borers that were harvested late, with inadequate residue management were at the highest risk, with an adjusted mean DON concentration of 1330 µg/kg. The combination of these three risk factors therefore multiplied the risk of DON contamination by 2.5 (Figure 2).

**Figure 3:**
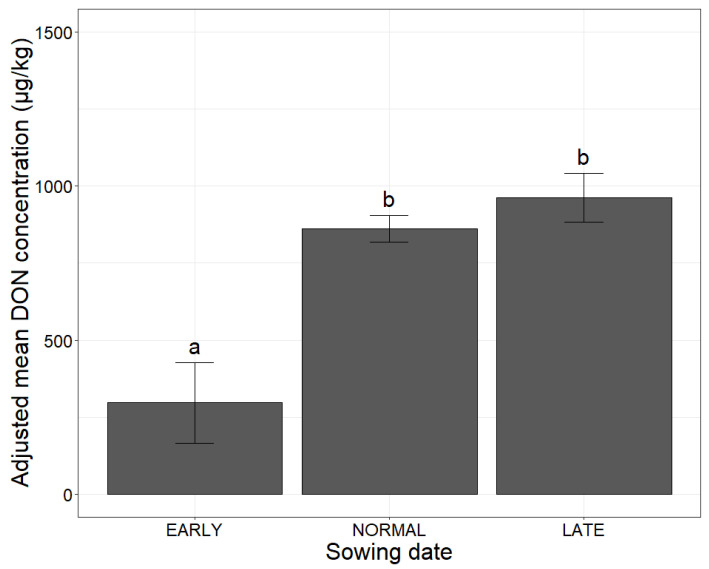
Mean adjusted DON concentration according to sowing date, in maize. The three categories of sowing date (EARLY, NORMAL, LATE) are shown on the *x*-axis. The adjusted mean DON concentration obtained with a mixed linear model (lmer(DON ~ sowing date + (1|Year))) applied to a database of 2027 observations is plotted on the *y*-axis. Different letters (a and b) above the bars indicate significant differences (*p*-value < 0.001) in ANOVA tests for each of the variables.

Late sowing significantly increased the adjusted mean DON concentration, by a factor of three relative to early sowing (Figure 3).

### 2.2. Association of Climatic Factors at Sensitive Periods Influencing DON Concentrations

This multiyear study of the effects of more than 450 climatic variables on DON contamination risk during the maize development cycle revealed a main effect of climate, particularly for temperature and humidity during three key periods: March, August and the two months immediately preceding harvest (*p*-value *<* 0.001 for both temperature and humidity, ANOVA). In France, March is the month before sowing (conservation of fungal inoculum on crop residues), August is the month in which maize post-flowering development occurs and the two months immediately preceding harvest correspond to the ripening period (*F. graminearum* growth and development during the two periods).

#### 2.2.1. Temperature and Humidity during the Pre-Sowing and Post-Flowering Periods

We calculated and compared the adjusted means for each category of mean maximum monthly temperature in March and humidity plot environment in August (Table 1).

**Figure 4:**
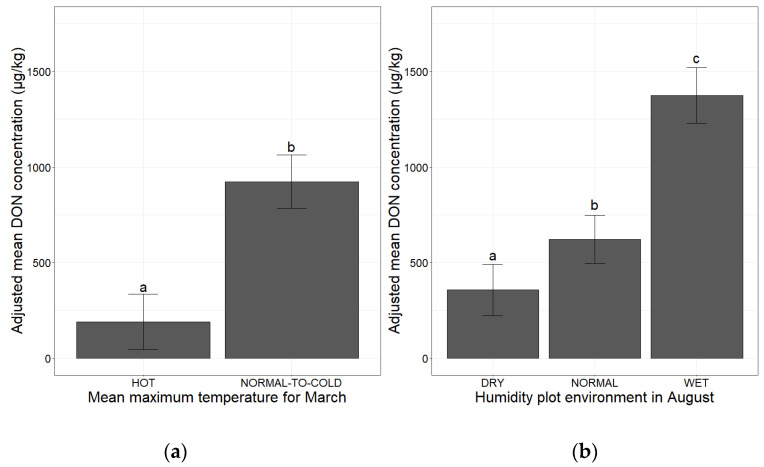
Mean adjusted DON concentration according to mean monthly temperature before sowing and after flowering. (**a**) Mean adjusted DON concentration according to mean maximum temperature in March; (**b**) mean adjusted DON concentration according to August humidity plot environment. The mean maximum monthly temperature category is indicated on the *x-*axis (HOT, NORMAL-TO-COLD). The humidity plot environment category is indicated on the *x-*axis (DRY, NORMAL, WET). The adjusted mean DON concentrations obtained by applying the mixed linear model (lmer(DON ~ March mean maximum monthly temperature/August humidity environment + (1|Year))) to a database of 2032 observations are plotted on the *y*-axis. Different letters (a, b and c) above bars indicate significant differences (*p*-value < 0.001) in ANOVA tests for each of the variables.

Mean maximum monthly temperature in March and humidity in August had significant effects on the risk of DON contamination in maize at the national scale in France (*p*-value < 0.001 for both climatic factors, ANOVA) (Table 1 and Figure 4). Lower DON concentration (divided by 8.5) was observed for a hot March, which corresponds to favorable temperature conditions for peritheces maturation, leading to earlier ascospores discharge, before maize flowering (Table 1 and Figure 4a). With August considered to be wet, favorable conditions for the fungal growth, DON concentration was multiplied by 3.7 relative to dry conditions (Table 1 and Figure 4b).

**Figure 5:**
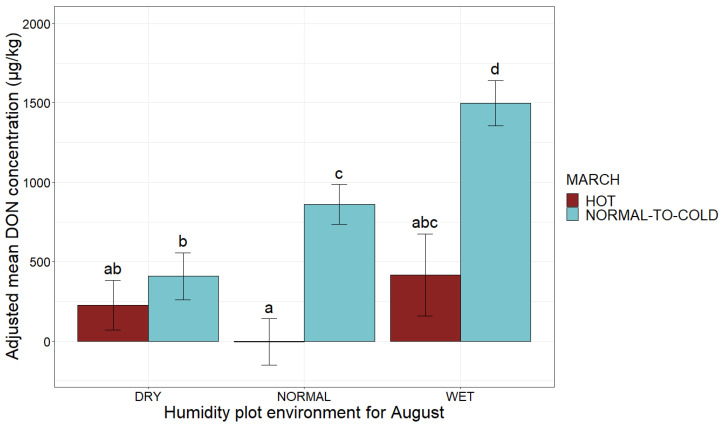
Adjusted mean DON concentration according to humidity plot environment at flowering and temperature before sowing. The humidity plot environment for August is shown on the *x*-axis (DRY, NORMAL, WET). The adjusted mean DON concentrations obtained by applying the mixed linear model (lmer(DON ~ August × March + (1|Year))) to a database of 2032 observations are shown on the *y*-axis. Blue bars correspond to observations for a normal-to-cold March, whereas red bars correspond to data for a hot March. Different letters above bars indicate significant differences (*p*-value < 0.05) in Tukey’s multiple comparison test.

The combination of the two climatic factors had a significant impact on DON contamination (R^2^ = 0.10, *p*-value < 0.001). Adjusted mean DON concentration increased as soon as the two factors were in the “at risk” category, and this increase was larger if both factors were in the “at risk” category (Figure 5). The plots with a wet August and normal-to-cold March were at the highest risk, these conditions multiplying the adjusted mean DON concentration by 6.6 (Figure 5).

#### 2.2.2. Characterization of a Hot End to the Maize Development Cycle

**Figure 6:**
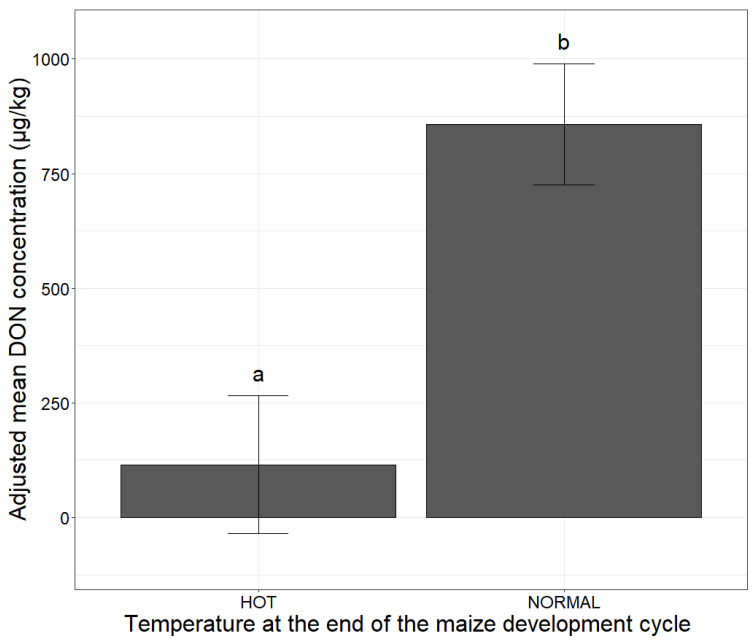
Adjusted mean DON concentration according to the presence or absence of a hot end to the maize development cycle. The presence or absence of hot conditions at the end of the cycle is indicated on the *x-*axis (HOT, NORMAL). The adjusted mean DON concentrations obtained by applying the mixed linear model (lmer(DON ~ end of cycle + (1|Year))) to a database of 2032 observations are plotted on the *y*-axis. Different letters (a and b) above bars indicate significant differences (*p*-value < 0.001) in ANOVA.

The individual effect of temperature at the end of the cycle, corresponding to grain filling and ripening in France, on the risk of DON contamination was significant (*p*-value < 0.001, ANOVA). Normal temperatures, favorable to fungal growth, at the end of the maize development cycle significantly increased the risk of DON concentration, multiplying the adjusted mean DON concentration by 7.5 (Table 2 and Figure 6).

**Figure 7:**
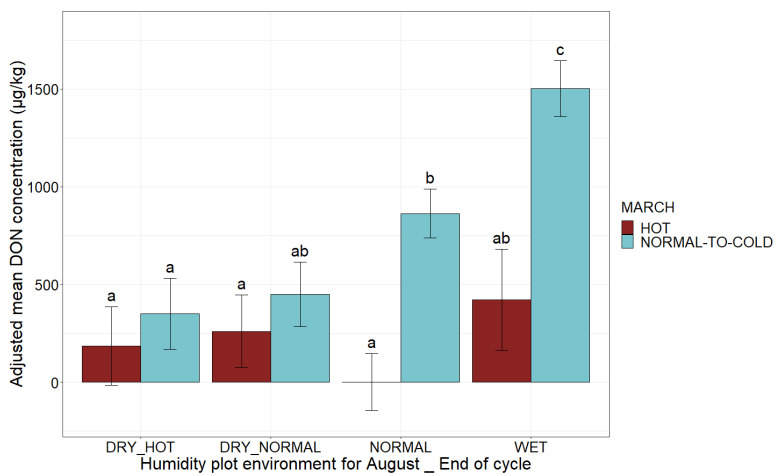
Mean adjusted DON concentration according to climatic conditions at the end of the maize development cycle, humidity during August and temperature during March. Climatic conditions at the end of the cycle and the humidity plot environment for August category are indicated on the *x-*axis (DRY_HOT, DRY_NORMAL, NORMAL, WET). The adjusted mean DON concentrations obtained by applying the mixed linear model (lmer(DON ~ August × March × End of cycle + (1|Year))) to a database of 2032 observations are plotted on the *y*-axis. Blue bars correspond to observations for a normal-to-cold March, whereas red bars correspond to data for a hot March. Different letters (a, b and c) above bars indicate significant differences (*p*-value < 0.05) in Tukey’s multiple comparison test.

The combination of a hot end to the maize development cycle and a dry August, unfavorable conditions for fungal growth in plants, slightly but not significantly decreased DON content by 1.4 (*p*-value > 0.05, test ANOVA, Figure 7). The combination of a hot end to the cycle and normal or wet weather during August were not encountered in our database.

### 2.3. Combinations of Categories for the Agronomic and Climatic Factors Can Create Definitions Determining Whether the EU Regulatory Limits for DON in Maize Are Respected

For a tool to be easy to use, the number of variables included must be limited. We chose not to retain sowing date, which is partly linked to harvest date, as the later the maize is sown, the later it is likely to be harvested.

**Figure 8:**
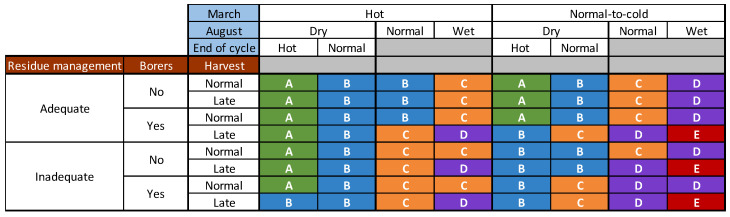
Grid based on the multiyear and national data illustrating the impact of combinations of agronomic and climatic risk factors on DON content in maize. Agronomic factors (residue management and borers) and harvest date are represented on the left of the grid. Climatic factors (mean monthly maximum temperatures in March, August humidity plot environment and temperature during the end of the cycle) are represented at the top of the grid. The possible associations were combined to create a common factor ITK. Their adjusted means were obtained by applying the mixed linear model (lmer(DON ~ ITK + (1|Year))) to a database of 2032 observations. These means were compared, grouped if not statistically different and then assigned to a DON risk class (A to E), as indicated by the different colors above. In ascending order, A corresponds to a very low risk (green), B to a low risk (blue), C to a moderate risk (orange), D to a high risk (purple) and E to a critical risk (red).

The possible associations between agronomic and climatic risk factors described above explained 14% of the variability of DON concentration observed over a period of 15 years at the national scale in France (R^2^ = 0.14, *p*-value < 0.001). The prevention grid indicates the possible combinations of the categories of these variables encountered in the multiyear and national database (Figure 8). Combinations between agricultural practices and climatic conditions with similar effects on DON contamination were grouped and ranked in five different groups: from A (very low risk) to E (critical risk) (Figure 8).

Class C, corresponding to a moderate DON risk, was the most represented class. From A to E, the risk gradually increased with combinations of different conducive agronomic and climatic risk factors (Figure 8). The high and critical risk classes corresponded to favorable climatic (i.e., normal-to-cold March and wet or normal August) factors combined with at least a late harvest and one of the other agronomic factors (the presence of borers and inadequate residue management) (Figure 8). In general, the presence of hot conditions at the end of the maize development cycle tended to decrease the risk class if August was considered “dry” (Figure 7).

**Figure 9:**
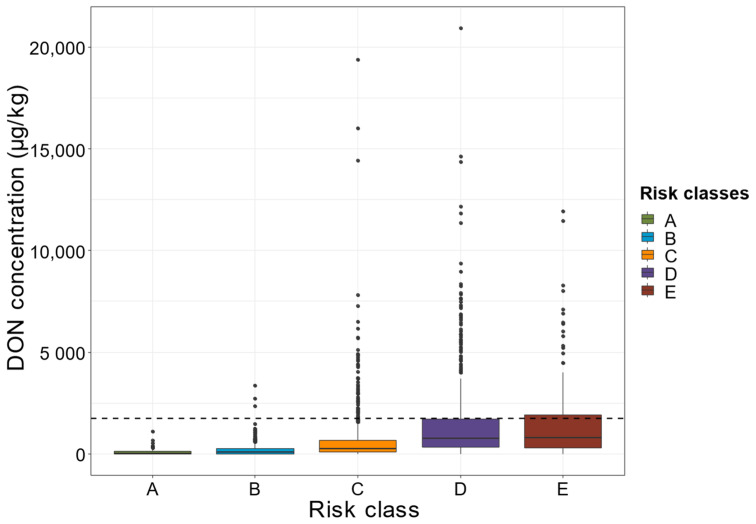
DON concentration according to risk class. The created DON risk classes are shown along the *x*-axis, in ascending order of risk, from left to right (A to E). The DON concentrations of the 2032 plots measured are shown on the *y*-axis. Colors distinguish the different risk classes: green for A, blue for B, orange for C, purple for D and red for E. The dashed line represents the maximum DON concentration authorized for maize for human consumption in the EU (1750 µg/kg).

The validation of this multiyear and national grid is illustrated in Figure 9. DON contamination was positively related to risk class (*p*-value < 0.001, ANOVA). Over the 15-year period for which data were analyzed, the distribution of DON values (mean, median, first and third quartiles) clearly increased from Class A to E (Figure 9). Extreme values were observed for each risk class (Figure 9). While 25% of the DON values for class D exceeded the maximum level of 1750 µg/kg allowed, this percentage increased to 31% for Class E (Figure 9).

The created DON risk classes accounted for 12% of the variability of DON concentration observed over the 15-year period (Table 3). This percentage fluctuated from year to year, from 0 to 40% (Table 3). The higher the percent, the more the risk classes explained the variability observed for annual DON concentrations. On the contrary, negative values, as in 2008, 2010 and 2017, indicated that the risk classes did not explain the trend of annual DON concentrations. In this latter case, this corresponded to harvest years with agronomic and climatic conditions concentrated in 2 or 3 consecutive risk classes, with often a large majority of plots assigned to 1 or 2 risk classes. The DON risk classes were consequently not significant.

## 3. Discussion

We identified and evaluated agronomic and climatic risk factors for DON contamination in the field with a 15-year database containing data from 2032 agricultural farm fields collected across all the French maize-growing regions. Such studies have been limited to date because preharvest strategies in maize fields mostly involve residue and pest management, fungicide application and the development of resistant hybrids [42]. Pest management was not included in the study due to the lack of information provided by farmers. In France, there is no Fusarium fungicide treatment on maize during cultivation for economic reasons. Most strategies for reducing DON risk in the field are based on the development of plants resistant to *F. graminearum* contamination and DON production [12,43]. In France, a varietal classification for DON susceptibility exists for maize, but the rate of variety turnover is very high in Europe, including France. Thus, many maize varieties are not evaluated for this criterion, which was therefore difficult to incorporate into our analysis without losing too many samples. GMOs are banned in France and cannot, therefore, be used as a tool for preventing DON contamination [13]. However, preventive measures that can be applied before and during the growth of the crop in the field are the first and foremost crucial step towards an effective integrated strategy for DON risk [14,44]. In this context, we provide a tool for the prevention of DON contamination based on the agronomic and climatic conditions encountered in French maize-growing areas (summarized in Figure 8). Each situation is associated with a DON risk class, from very low to critical.

We identified the presence of borers, residue management and sowing and harvest dates as agronomic factors associated with DON contamination. Several studies have already evaluated them as risk factors for preharvest DON contamination [15,16,21,22,27,28,30]. In particular, residue management, harvest dates and the presence of borers were included in a previous prevention matrix created by Arvalis-Institut du végétal in 2007 [24]. Some preharvest strategies have already been proposed, based on the use of agronomic practices related to lower DON contamination, such as the use of an appropriate selection of maize hybrids, appropriate residue management and avoiding late sowing and harvest dates, for example [22,30,41,45]. All of these proposals were developed on the basis of field experiments. In our study, we evaluated these factors in real production conditions, at a nationwide scale. Only three agronomic practices were identified as significantly associated with the risk of contamination: the presence of borers, sowing and harvest dates. Given the strong correlation between these last two variables, we decided to choose only the harvest date, most cited in the literature, to avoid complicating our prevention tool. In our study, residue management slightly increased, but not significantly, DON risk. The small number of plots with residue management considered as “inadequate” may not be large enough (20% of the data) to observe statistical differences. These agronomic risk factors explain a very small part (2%) of the variability of DON data around its average observed over 15 years at the national scale in France, compared to climatic conditions (11%). However, the part of the variability explained by all the agronomic factors does not prejudge the significance of their relationship with the DON content.

The climate is often unpredictable and difficult to modulate as a prevention tool. However, three critical periods before, during and at the end of the maize growing season favoring *F. graminearum* germination, infection, growth and DON contamination were identified and studied in detail: pre-sowing, flowering and kernel drying. Temperature influences the development of *F. graminearum* perithecia on crop residues [31]. Perithecia developed at temperatures between 12 and 28 °C, and maturation occurred only under warm conditions, around 16, 20 and 24 °C [31]. In France, March corresponds to the month before sowing. Warmer monthly conditions may create favorable conditions for an early fungus germination without plant hosts to infect. The rate of available inoculum potential can then be lowered for subsequent contaminations. High level of humidity increase *F. graminearum* infection rate, especially during the sensitive period of flowering corresponding to July in France [37,38]. This ambient humidity may facilitate post-contamination fungal growth by creating a favorable environment [46], for example, during the post-flowering period corresponding to August in France. Humidity may determine the ability of the fungus to grow after contamination and, consequently, also its ability to produce DON. *F. graminearum* grows best at temperatures between 24 and 26 °C [10,34], whereas *Fusarium verticillioides* continues to grow at temperatures above 28 °C [47]. A dry period with high temperatures before and during grain filling favors ear infection with *F. verticillioides* and *F. temperatum*, whereas the frequency of *F. graminearum* is higher at lower temperatures and in humid conditions [48]. A complex combination of competitive (*F. graminearum* was outcompeted in mixed inoculations) and facilitative (infection by *F. verticillioides* was facilitated by prior infection with *F. graminearum*) interactions shapes the *F. graminearum*–*F. verticillioides* community in maize [49]. In this context, higher temperatures in the two months immediately preceding harvesting, corresponding to the kernel drying period, may increase the levels of infection with other fungi at the expense of *Fusarium graminearum*. Our findings confirm the influence of temperature and moisture conditions on DON contamination during these three key periods of the maize development cycle. We went further, by defining the thresholds above which monthly temperatures and humidity in France can be considered “hot” and “wet”, respectively. Complicated climatic variables were simplified through their transformation into more easily usable variables. Our findings confirm the climatic and agronomic risk factors identified in previous studies. However, we went further by (1) constructing simple, easy-to-use agronomic and climatic explanatory variables and (2) creating a preharvest tool for the prevention of DON contamination in the field.

The national multiyear grid ranks associations between agronomic and climatic conditions from very low to critical in terms of DON contamination risk in French maize-growing areas. Several tools have been created for estimating the risk of DON contamination in wheat, but few such tools exist for maize [44,50]. This may be due to the greater variability of the silking period in maize, or the strong relationship between contamination and insect damage [44,51]. However, in the first decade of this century, a European tool was created to help farmers, agricultural cooperatives and processors to manage the DON contamination of silage maize in the Netherlands. Asselt et al. [51] developed a mechanistic model describing fungal infection and subsequent growth and the formation of DON in the Netherlands. Exclusively on the basis of climatic factors (temperature, rainfall, wind speed and relative humidity), the authors were able to classify the various years studied in terms of the risk of DON contamination, from a low to critical DON risk, for farm fields in the Netherlands from 2002 to 2007 [51]. The authors confirmed the major contribution of humidity and temperature conditions during flowering and later in the growing season to fungal growth and DON contamination in maize [51]. Temperature before sowing, humidity during post-flowering and temperature at the end of the maize development cycle provide less information about the climatic conditions than the factors included in this tool. We selected these three factors because they appeared to be the most easily understandable for use in an educational tool. Indeed, other agronomic factors, such as sowing date, and climatic factors were also found to have a significant effect on DON concentration. However, our target was to create an easy-to-use tool for farmers and agricultural cooperatives, and we therefore had to make choices about which variables to retain and which to discard. If our target had been to create a forecasting model, we would have considered a combination of all the significant agronomic and quantitative climatic factors. In our study, we made compromises to meet the expectations of the French agricultural sector. Despite the good results obtained, Asselt et al. pointed out that their tool had several limitations, including a lack of information about agricultural practices [51]. Notably, information about rotation and tillage were absent and would need to be included in further developments of tools of this type [51]. The authors considered insect damage to be absent due to their low abundance of borers in the Netherlands. However, this factor may need to be included in the future following increases in borer abundance due to climate change. Hooker et Schaafsma [52], with a database over 7 years in Ontario, observed that the effect due to year (or to weather, perhaps) accounted for 12% (*p*-value < 0.0001) of the variation in concentration of DON in maize, similar to the 11% for climatic conditions found in our study. By adding the hybrid factor, they created a prevention tool explaining 42% of DON variability over the 7 years. Our selection of variables accounts for 14% of the variability over the whole of France over a period of 15 years. One reason for this low percentage could be the existence of other factors not considered in our study but with a significant role, such as hybrid susceptibility [52]. Compared to existing models, the particularities of our tool are as follows: (1) a tool created over a 15-year study at a national scale, taking into account both changes in climate and agricultural practices; (2) using easy-to-target agronomic practices for farmers; (3) qualitative climatic factors adaptable by farmers according to their plot conditions; (4) estimation of the DON risk class before harvesting to adapt possible management.

The prevention tool assigned a risk class to each farm field over 15 years at the national scale. Differences in the significance of the risk class were observed from year to year. The number of observations differed between years, with more than 100 agricultural plots observed in some years, and fewer than 30 in others. The four risk classes also differed in terms of their representativeness, with the possible overrepresentation of certain risk classes at the expense of others. The prevention tool was developed and validated with data and information from agricultural farm fields in the various French maize-growing regions. The same approach has already been used to create a similar prevention tool for fumonisins (FUMO), mycotoxins produced by *Fusarium verticillioides*, in maize [53]. As with the tool created for FUMO, climatic quantitative variables were transformed into climatic qualitative ones, to facilitate their use by farmers who can adapt them to their own climatic conditions. Indeed, farmers characterize themselves if temperatures are considered as “hot” or “normal-to-cold” and if humidity is “dry” or “wet”. Farmers and agricultural cooperatives manage the variables themselves to correspond to their own field realities, which should help to reduce regional effects. While the climatic conditions favoring *F. verticillioides* growth and contamination are drought and heat, mild and humid conditions are highlighted for *F. graminearum* [10,31,54]. The presence of borers is related to higher DON and FUMO content, which highlights the importance of this factor for mycotoxin contamination in maize. For both tools, the association of a risk class with each combination of simplified categories of the factors considered explained between 10% and 12% of the variability observed for the two mycotoxins over the 15 years studied. This proportion decreases with the number of possible associations of risk factors (from 64 to 5, for example, for DON) due to a loss of information. The number of categories was decreased to combine similar field situations and to create an easier tool to interpret. In the two studies, although most of the plots were well-characterized over the 15-year period, some were considered to be at low-to-medium risk of FUMO or DON contamination while their mycotoxin concentrations exceeded the regulatory thresholds. One reason is probably the broader nature of the situations, based on the grouping together of different agronomic and environmental conditions into the same risk class. Our decision to decrease the number of categories had the consequence of decreasing the amount of variability explained, potentially leading to an underestimation of DON risk in some plots. As EU regulations for mycotoxins are continually tightened, the development and use of accurate tools for preventing the contamination of batches of maize grain will be essential. The good results obtained with this tool for DON, and with the previous tool for FUMO [53], for the 15-year period studied at nationwide level in France suggest the development of a similar approach in other countries and for other mycotoxins.

One perspective of this work is the possibility of extending the use of this tool to other EU countries. However, there are already large differences in systems and environments at the national scale in France and these differences would be even larger between different European countries. Jajić et al. [55] compared the levels of maize contamination with DON between several Eastern European countries and concluded that the observed differences were potentially related to specific agricultural factors and climatic conditions. The use of the prevention tool in a region or country other than that in which it was created would require an understanding of its regional component and adaptation of the tool to correspond to new regional realities [50]. In that context, our DON risk grid would be difficult to apply in its current state in other European countries, but it could be modified to deal with other field realities. The development of a similar approach could be used to select appropriate agronomic practices and meteorological conditions during the plant sensitive periods. The grid could also be fortified by considering the co-contamination of maize with other mycotoxins. Indeed, different fungi can co-contaminate the same maize plant and produce different mycotoxins, such as DON (produced by *Fusarium graminearum*) and fumonisins (produced by *Fusarium verticillioides*). Scientific interest in the biological effects of mycotoxin mixtures is increasing. Further studies on the nature of the relationship between the two fungi, and between their mycotoxins, are required [49,56]. *F. graminearum* and *F. verticillioides* can occur together and produce mycotoxins on the same plant following artificial infections, but the type of interaction may depend on weather conditions [49,56,57]. In the challenging scenario of climate change, the co-occurrence of mycotoxins in maize is problematic for the creation of accurate prevention tools, as the presence of one mycotoxin may affect the production of other mycotoxins. Further studies are required to incorporate this balance between co-contaminants into the prevention tool.

## 4. Conclusions

A national multiyear field survey was performed in French maize-growing areas, to study the relationship between DON contamination and agricultural practices and climatic conditions. Several agronomic and climatic factors were highlighted. However, for the creation of an easy-to-use tool for farmers and agricultural cooperatives, we selected the most understandable factors. We retained the following agronomic factors: the presence/absence of borers, a normal/late harvest date and adequate/inadequate residue management. We considered climatic conditions during three sensitive stages of maize development: temperature before sowing, humidity post-flowering and the presence of a hot end to the maize development cycle with a dry August. The effects of their various categories were evaluated, and the possible combinations of these categories with similar effects on DON content were grouped and ranked, to classify the risk in the field from very low to high. The riskiest situation was the combination of inadequate residue management with a late harvest date and the presence of borers, in farm fields with “normal-to-cold” temperatures in March and damp conditions in August. These risk factors have been highlighted in several studies, individually or in combination, but not all five together. Our DON prevention tool includes agronomic practices and climatic conditions during sensitive periods. The transformation of quantitative climatic variables into qualitative variables enabled the integration of easy-to-use climatic sequences into a field tool created for all stakeholders in the sector. The different realities of French maize-growing areas were integrated into a single prevention tool. A better understanding of the biotic relations between co-contaminants will make it possible to determine the balance between mycotoxins, for incorporation into future preventive tools.

## 5. Materials and Methods

### 5.1. Multiyear Field Surveys at the French National Scale

Maize is the second most produced cereal crop in France, with an annual mean area of 1.5 million hectares sown. With our national mycotoxin monitoring program for the maize harvest in France, we collected 2032 samples from 2032 maize fields at harvest from 2004 to 2020 (Table 4). We issued a call for volunteers among farmers in French maize-growing areas. On a multiyear basis over the entire study, the number and choice of farm fields included (1) the relative importance of each area to French maize production and (2) the relative importance of maize to French cereal production. The different number of farm fields studied each year depended on the annual internal budget available to fund this study: high enough for more than 100 samples (2018, 2019, etc.) and too low in some years to have any samples (2013, 2014) (Table 4).

**Figure 10:**
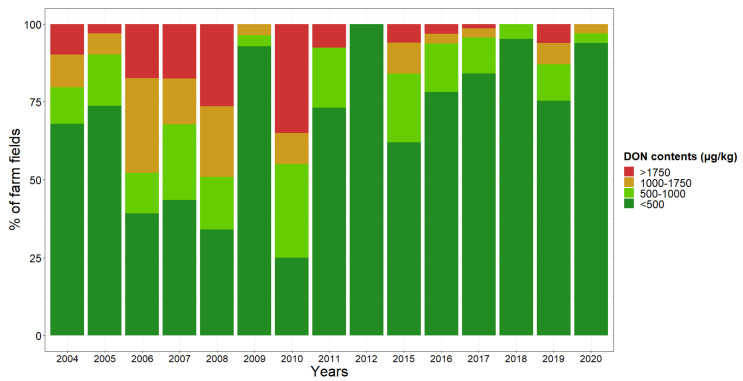
Annual percentage of farm fields into DON concentration classes. Years are shown in chronological order. Four different concentration classes were specified: <500 µg/kg (dark green), 500–1000 µg/kg (light green), 1000–1750 µg/kg (orange) and >1750 µg/kg (red). The number of samples per year is indicated in Table 4.

The annual variations in the measured DON contents are summarized in Figure 10. Over the 15-year period, DON concentrations varied from high in some years (2006, 2008, etc.) to very low in others (2009, 2018, etc.).

**Figure 11:**
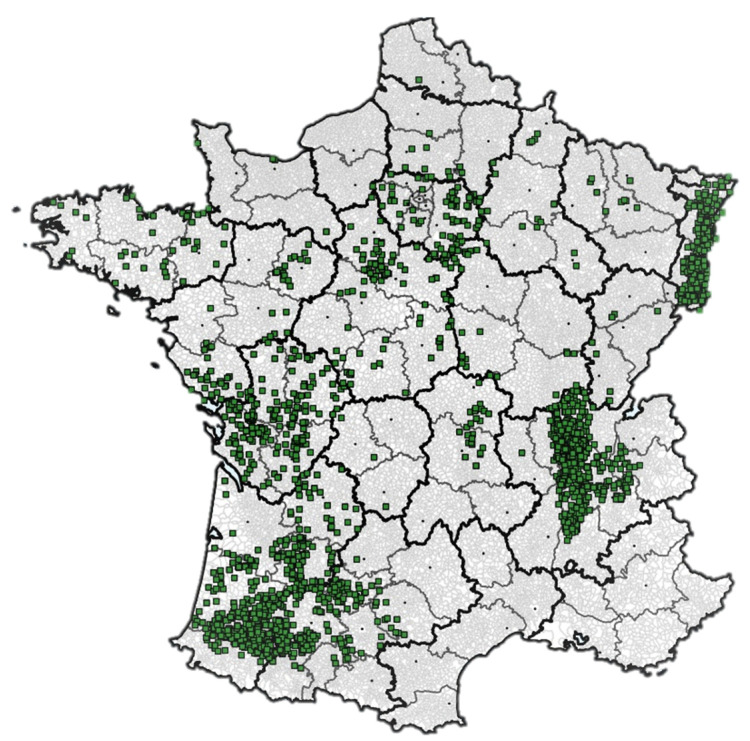
Geographic distribution of field samples in France over the study period.

The localization of the farm fields is illustrated in Figure 11. The spatial distribution, similar in all 15 years of the study, is representative of the main French maize-growing areas.

### 5.2. Sample Collection

At harvest, all farmers collected and prepared samples according to the following protocol: (a) avoid sampling field margins, (b) avoid static grain sampling and (c) sample moving grains during three different periods of emptying of the combine harvester. During harvest, three different subsamples of at least 1 kg were, therefore, collected manually from the moving grains. A 3 kg sample was finally obtained by combining these three subsamples for each farm field.

### 5.3. Sample Preparation for Analysis

A laboratory cleaner and separator (MINI-PETKUS 100 and 200, PETKUS Technologie GmbH, Rohr, France) removed all impurities from the kernels to obtain cleaned grain samples. A quantity of 1.5 kg of homogeneous sample was then selected for analysis. This sample was ground in a laboratory hammer mill fitted with a 1 mm screen (TITAN 2000, F.A.O., Vitré, France).

### 5.4. Deoxynivalenol Quantification

From 2003 to 2012, two accredited French laboratories analyzed DON by liquid chromatography with photometric detection (HPLC-UV). Samples were randomly shipped to either laboratory. The first laboratory took the limit of detection of 20 µg/kg, and the corresponding limit of quantification was 80 µg/kg. The second laboratory took the limit of detection of 45 µg/kg, and the corresponding limit of quantification was 183 µg/kg.

From 2015 to 2020, DON concentration was determined by liquid chromatography–tandem mass spectrometry at the second accredited laboratory in France. The limit of detection was 10 µg/kg, and the corresponding limit of quantification was 25 µg/kg.

In our study, for all DON contents below the limits of detection or quantification, we assigned a value corresponding to half of each limit. The change in these limits therefore had no impact since the DON levels concerned stay low, and all values were retained. Likewise, changes in the analysis method used have no impact because they are reference methods whose accuracy is checked by COFRAC accreditation.

### 5.5. Agronomic Factors

Each farmer completed a questionnaire developed by Arvalis-Institut du végétal with items concerning several agronomic parameters from sowing to harvest (including, in particular, residue management, sowing and harvest dates and whether or not borer insects were present on the ear or on the stem, etc.), location and soil type.

**Figure 12:**
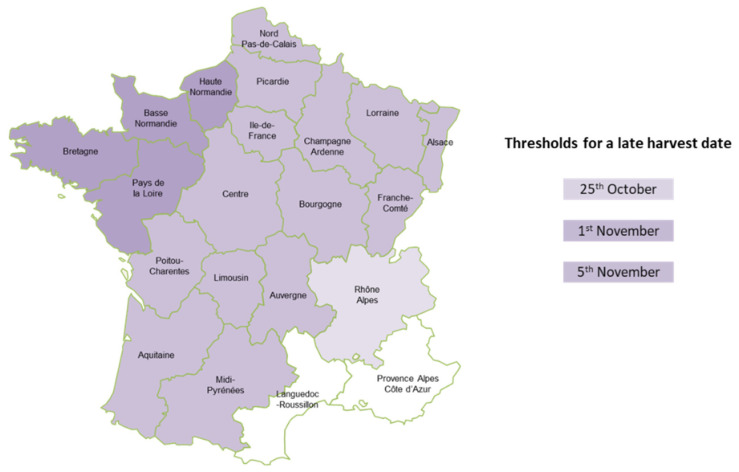
*Département*-level thresholds for a late harvest date.

For each year, farmers indicated whether the sowing date was early, normal or late. Late harvests were defined using thresholds defined at the *département* (a French administrative unit similar to a county) level (Figure 12). These thresholds were chosen to correspond to the typical seasonal dates. Fields harvested before this limit are classified in the “normal” category and fields harvested after this limit correspond to late harvests.

Residue management was assessed on the basis of three items of information: previous crop, plowing and the crushing of residues (factors highlighted in both literature and field expertise). It was considered adequate (related to lower DON concentration) if there was plowing or if the residues were at least crushed with a previous crop that was neither maize nor sorghum (Table 5).

### 5.6. Climatic Factors

#### 5.6.1. Quantitative Climatic Parameters

Spatialization of each farm field based on its Lambert coordinates, obtained with the town and zip code, was used to get meteorological data from the nearest weather station (Arvalis-Institut du végétal or Météo France). Nearly 700 weather stations, distributed throughout mainland France, were used to obtain spatialized climatic data. These data were then converted into daily parameters, used to obtain the studied weather variables [58].

Based on field expertise and previous studies, we selected more than 450 parameters to study, related to temperature, rainfall, and frost days, for example. The climatic conditions targeted were those considered favorable for the development of *F. graminearum*, but they were also linked to a greater sensitivity of the maize plant. These variables were calculated over specific periods of the plant cycle: calendar (March, August, etc.) or in relation to sensitive stages, such as flowering (date estimated by our internal phenological models).

#### 5.6.2. Quantitative Climatic Parameters into Single Qualitative Variables

For the four selected quantitative climatic parameters, we transformed them into qualitative single variables.

One temperature threshold, the third quartile of all the values obtained in France over a period of 15 years, was used to transform the quantitative variables for March into a single qualitative variable (Table 6). The two categories differentiating a “hot” month from a “normal-to-cold” month were defined on the basis of their range of values for seasonal climatic variables (Table 6). Two thresholds, the first and third quartiles of all the values obtained in France over a period of 15 years, were used to transform the quantitative variables for August into a single qualitative variable (Table 6). The three categories differentiating a “dry” month from a “normal” or “wet” month were defined on the basis of their range of values for seasonal climatic variables (Table 6).

For the temperature 30–60 days before harvest and the number of days above 28 °C, 0–30 days before harvest, we used the third quartiles of all the values measured throughout France over a period of 15 years as thresholds to transform these quantitative variables into qualitative variables (Table 7). These two variables were then combined to form a temperature variable for the end of the maize development cycle: end of cycle, with “hot” and “normal” as possible values (Table 7).

### 5.7. Statistical Analyses

#### 5.7.1. Selection of Climatic and Agronomic Risk Factors

A least absolute shrinkage and selection operator (LASSO) regression was used to get a first shortlist of climatic risk factors with a constraint of type L1 [59,60]. A random forest approach then made it possible to keep the most relevant variables [61].

With one linear mixed model, we analyzed individual and combined effects of the selected quantitative climatic factors and agronomic variables on DON content (as fixed effects), and year as a random effect. We performed analyses of variance (ANOVA) with the car package for R [62] to identify the significant risk factors.

#### 5.7.2. Conversion of Climatic Quantitative Factors into Categorical Variables

The distribution of values for the four quantitative climatic factors selected over a period of 15 years was used to transform them into three qualitative variables (see Section 5.6.2). Each factor was first converted into qualitative variables with four balanced categories determined by its first, second and third quartiles. Four linear mixed models were used to test their individual effects on DON contamination (as fixed effects), with year as a random effect. We performed pairwise comparisons between the four categories, based on Tukey-adjusted least-squares means, with p-values < 0.05 considered significant, with the multcomp package for R [63]. We grouped the categories, which were not significantly different, to create two dichotomous variables (March and End of cycle) and one variable with three categories (August), with the third or both the first and the third as cutoffs.

#### 5.7.3. Univariate and Multivariate Analyses

Univariate analyses were first performed on the selected agronomic and categorical climatic factors, to investigate their individual effects on DON content (Table 8). We then performed multivariate analyses to test the effects of combinations of risk factors on DON content (Table 8). Combinations of agricultural practices and climatic conditions with effects on DON contamination that were not significantly different were grouped and associated with a DON risk class tested by univariate analysis (Table 8).

With a linear mixed-model approach, we first analyzed individual risk factors and combinations of risk factors, with agronomic and/or climatic factors as fixed effects and year as a random effect (Table 8). Several analyses of variance (ANOVA), with the *car* package of R, were performed to assess the effects of individual risk factors and combinations of risk factors on DON content. We then calculated and analyzed the adjusted means with the emmeans package of R [64]. Finally, we evaluated with pairwise comparisons, based on Tukey-adjusted least-squares means with *p*-values < 0.05 considered significant, the statistical significance of differences between the categories of a risk factor, and between combinations of categories of different risk factors, using the multcomp package for R [63].

All data processing and statistical analyses were performed with R, version 4.0.2 (R Development Core Team, 2020) [65].

## Figures and Tables

**Table 1:** Adjusted Mean DON Concentrations for Each Category of the Two Selected Climatic Factors.

Climatic Factors	Possible Values	Adjusted Mean DON Concentration (µg/kg)	Comparison of Means
March, mean maximum monthly temperature	Hot	103	a ^1^
	Normal-to-cold	878	b ^1^
August, humidity plot environment	Dry	365	a ^2^
	Normal	616	b ^2^
	Wet	1343	c ^2^

^1^ Mean values in each row followed by different letters are significantly different (*p*-value < 0.001; ANOVA); ^2^ The same letters are used as for 1, but they do not correspond to the same groups.

**Table 2:** Adjusted mean DON concentrations at the end of the maize development cycle.

End of Cycle	Adjusted Mean DON Concentration (µg/kg)	Comparison of Means
Hot	115	a ^1^
Normal	858	b ^1^

^1^ The mean values in each row followed by different letter are significantly different (*p*-value < 0.001; ANOVA).

**Table 3:** Significance of the DON risk classes created and percentage of farm fields for each risk level, for each year studied. For each year, *p*-values and R^2^ are provided for the combinations of risk factors in linear fixed-effect models with risk class as the fixed effect.

Harvest Year	*n*	*p*-Values	R^2^	Percentage of Farm Fields for Each Risk Level				
				A	B	C	D	E
2004	265	<0.001	0.06	3	3	25	41	28
2005	426	<0.001	0.08	1	31	52	14	2
2006	441	<0.001	0.13	0.5	14	32.5	48	5
2007	177	>0.05	0.02	0	0	24	73	3
2008	53	>0.05	−0.008	0	2	38	51	9
2009	28	>0.05	0.01	36	53	11	0	0
2010	20	>0.05	−0.01	5	0	85	10	0
2011	26	<0.01	0.43	4	31	58	7	0
2012	13	NA	NA	0	69	31	0	0
2015	50	<0.001	0.26	0	6	64	26	4
2016	64	<0.001	0.28	23	45	27	5	0
2017	69	>0.05	−0.003	10	70	20	0	0
2018	125	>0.05	0.04	47	18	32	3	0
2019	146	<0.001	0.10	15	46	32	6	1
2020	129	<0.001	0.1	50	18	27	5	0
2004–2020	2032	<0.001	0.12	9	21	35	29	6

**Table 4:** Sampling for the field survey, by year.

Years	Samples
2004	265
2005	426
2006	441
2007	177
2008	53
2009	28
2010	20
2011	26
2012	13
2013	0
2014	0
2015	50
2016	64
2017	69
2018	125
2019	146
2020	129

**Table 5:** Qualification of residue management based on previous crop, the crushing of residues and plowing.

Previous Crop	Plowing	Crushing of the Residues	Residue Management
Others	Yes	Yes	Adequate
		No	Adequate
	No	Yes	Adequate
		No	Inadequate
Maize, Sorghum	Yes	Yes	Adequate
		No	Adequate
	No	Yes	Inadequate
		No	Inadequate

**Table 6:** Transformation of selected quantitative climatic parameters into qualitative variables.

Quantitative Climatic Factors	Thresholds	Qualitative Climatic Factors	Possible Values
March, mean maximum monthly temperature	>14.5 °C	March, mean maximum monthly temperature	Hot
	<14.5 °C		Normal-to-cold
August, (Rain–ETP ^1^)	<−93.6	August, humidity plot environment	Dry
	−93.6< … <−16.8		Normal
	>−16.8		Wet

^1^ ETP is evapotranspiration.

**Table 7:** Combination of the two quantitative variables during the 1–2 months preceding harvest into a single qualitative variable.

30–60 Days before Harvest, Mean Maximum Monthly Temperature	0–30 Days before Harvest, Number of Days with a Mean Maximum Daily Temperature Above 28 °C	End of Cycle
>26 °C	>3	Hot
>26 °C	<3	
<26 °C	<3	Normal
<26 °C	>3	

**Table 8:** The linear mixed model approach for testing the individual and combined effects of risk factors on DON content. The models are ordered according to the different stages of the process.

Linear Mixed Models	Objectives
[DON] = Agro_i_^1^/Clim_j_^2^ + (1|Year)	Individual effect
[DON] = ∑(Agro_i1_/Clim_j1_ × Agro_i2_/Clim_j2_) + (1|Year)	Combination of climatic or agronomic effects
[DON] = ∑(Agro_i_ × Clim_j_) + (1|Year)	Combination of agronomic and climatic effects
[DON] = Risk classes + (1|Year)	Individual effect of the DON risk class

^1^ Agro_i_ is one of the selected agronomic variables (harvest date, residue management and presence of borers). ^2^ Clim_j_ is one of the selected climatic variables (March, August and end of the maize development cycle).

## Data Availability

The data presented in this study are available on request from the corresponding author. The data are not publicly available because they were collected privately by Arvalis-Institut du végétal.

## References

[1] La Filière Maïs, Maïserie et Maïs Doux en Chiffres. https://www.passioncereales.fr/dossier-thematique/la-fili%C3%A8re-mais-maiserie-et-mais-doux-en-chiffres.

[2] Logrieco A., Bottalico A., Mulé G., Moretti A., Perrone G., Xu X., Bailey J.A., Cooke B.M. (2003). Epidemiology of Toxigenic Fungi and Their Associated Mycotoxins for Some Mediterranean Crops. Epidemiology of Mycotoxin Producing Fungi.

[3] Sobrova P., Adam V., Vasatkova A., Beklova M., Zeman L., Kizek R. (2010). Deoxynivalenol and Its Toxicity. Interdiscip. Toxicol..

[4] Vigier B., Reid L.M., Dwyer L.M., Stewart D.W., Sinha R.C., Arnason J.T., Butler G. (2001). Maize Resistance to Gibberella Ear Rot: Symptoms, Deoxynivalenol, and Yield1. Can. J. Plant Pathol..

[5] Pestka J.J., Smolinski A.T. (2005). Deoxynivalenol: Toxicology and Potential Effects on Humans. J. Toxicol. Environ. Health Part B.

[6] Neme K., Mohammed A. (2017). Mycotoxin Occurrence in Grains and the Role of Postharvest Management as a Mitigation Strategies. A Review. Food Control.

[7] Srecec S. (2013). Decreasing Deoxynivalenol Concentration in Maize within the Production Chain of Animal Feed. Agro Food Ind. Hi Tech.

[8] Xu Y., Huang Z.-B., He Q.-H., Deng S.-Z., Li L.-S., Li Y.-P. (2010). Development of an Immunochromatographic Strip Test for the Rapid Detection of Deoxynivalenol in Wheat and Maize. Food Chem..

[9] Richard J.L. (2007). Some Major Mycotoxins and Their Mycotoxicoses—An Overview. Int. J. Food Microbiol..

[10] Munkvold G.P., Xu X., Bailey J.A., Cooke B.M. (2003). Epidemiology of Fusarium Diseases and Their Mycotoxins in Maize Ears. Epidemiology of Mycotoxin Producing Fungi.

[11] Quesada-Ocampo L.M., Al-Haddad J., Scruggs A.C., Buell C.R., Trail F. (2016). Susceptibility of Maize to Stalk Rot Caused by *Fusarium Graminearum* Deoxynivalenol and Zearalenone Mutants. Phytopathology.

[12] Gaikpa D.S., Miedaner T. (2019). Genomics-Assisted Breeding for Ear Rot Resistances and Reduced Mycotoxin Contamination in Maize: Methods, Advances and Prospects. Appl. Genet..

[13] Kuntz M. (2014). The GMO Case in France: Politics, Lawlessness and Postmodernism. GM Crops Food.

[14] Munkvold G.P., Arias S., Taschl I., Gruber-Dorninger C., Serna-Saldivar S.O. (2019). Chapter 9-Mycotoxins in Corn: Occurrence, Impacts, and Management. Corn.

[15] Ostry V., Ovesna J., Skarkova J., Pouchova V., Ruprich J. (2010). A Review on Comparative Data Concerning Fusarium Mycotoxins in Bt Maize and Non-Bt Isogenic Maize. Mycotoxin Res..

[16] Papst C., Utz H.F., Melchinger A.E., Eder J., Magg T., Klein D., Bohn M. (2005). Mycotoxins Produced by Fusarium Spp. in Isogenic Bt vs. Non-Bt Maize Hybrids under European Corn Borer Pressure. Agron. J..

[17] Miller J.D., Miller J.D., Trenholm H.M. (1994). Epidemiology of *Fusarium graminearum* diseases of wheat and corn. Mycotoxins in Grain: Compounds Other than Aflatoxin.

[18] Folcher L., Weissenberger A., Delos M. (2012). Quantitative Relationships between Ostrinia Nubilalis Activity and Deoxynivalenol Contamination in French Maize. Int. J. Pest. Manag..

[19] Agusti N., Bourguet D., Spataro T., Délos M., Eychenne N., Folcher L., Arditi R. (2005). Detection, Identification and Geographical Distribution of European Corn Borer Larval Parasitoids Using Molecular Markers. Mol. Ecol..

[20] Schaafsma A.W., Hooker D.C., Baute T.S., Illincic-Tamburic L. (2002). Effect of *Bt* -Corn Hybrids on Deoxynivalenol Content in Grain at Harvest. Plant Dis..

[21] Lauren D.R., Smith W.A., Di Menna M.E. (2007). Influence of Harvest Date and Hybrid on the Mycotoxin Content of Maize (*Zea Mays*) Grain Grown in New Zealand. N. Zeal. J. Crop Hortic. Sci..

[22] Blandino M., Reyneri A., Vanara F., Tamietti G., Pietri A. (2009). Influence of Agricultural Practices on *Fusarium* Infection, Fumonisin and Deoxynivalenol Contamination of Maize Kernels. World Mycotoxin J..

[23] Eckard S., Wettstein F.E., Forrer H.-R., Vogelgsang S. (2011). Incidence of Fusarium Species and Mycotoxins in Silage Maize. Toxins.

[24] Orlando B., Méléard B., Coudure R. (2015). Qualité sanitaire du maïs grain: La prévention pour mieux maîtriser les risques. Perspect. Agric..

[25] Champeil A., Doré T., Fourbet J.F. (2004). Fusarium Head Blight: Epidemiological Origin of the Effects of Cultural Practices on Head Blight Attacks and the Production of Mycotoxins by Fusarium in Wheat Grains. Plant Sci..

[26] Pereyra S.A., Dill-Macky R., Sims A.L. (2004). Survival and Inoculum Production of Gibberella Zeae in Wheat Residue. Plant Dis..

[27] Blandino M., Pilati A., Reyneri A., Scudellari D. (2010). Effect of Maize Crop Residue Density on Fusarium Head Blight and on Deoxynivalenol Contamination of Common Wheat Grains. Cereal. Res. Commun..

[28] Oldenburg E., Brunotte J., Weinert J. (2007). Strategies to Reduce DON Contamination of Wheat with Different Soil Tillage and Variety Systems. Mycotox Res..

[29] Champeil A., Fourbet J.F., Doré T., Rossignol L. (2004). Influence of Cropping System on Fusarium Head Blight and Mycotoxin Levels in Winter Wheat. Crop Prot..

[30] Maiorano A., Blandino M., Reyneri A., Vanara F. (2008). Effects of Maize Residues on the Fusarium Spp. Infection and Deoxynivalenol (DON) Contamination of Wheat Grain. Crop Prot..

[31] Manstretta V., Rossi V. (2016). Effects of Temperature and Moisture on Development of Fusarium *Graminearum gerithecia* in Maize Stalk Residues. Appl. Environ. Microbiol..

[32] Sutton J.C. (1982). Epidemiology of Wheat Head Blight and Maize Ear Rot Caused by *Fusarium graminearum*. Can. J. Plant Pathol..

[33] Tschanz A.T., Horst R.K., Nelson P.E. (1976). The Effect of Environment on Sexual Reproduction of Gibberella Zeae. Mycologia.

[34] Booth C. (1971). The Genus Fusarium. Genus Fusarium.

[35] Mansfield M.A., De Wolf E.D., Kuldau G.A. (2005). Relationships Between Weather Conditions, Agronomic Practices, and Fermentation Characteristics with Deoxynivalenol Content in Fresh and Ensiled Maize. Plant Dis..

[36] Munkvold G.P., Hellmich R.L., Showers W.B. (1997). Reduced Fusarium Ear Rot and Symptomless Infection in Kernels of Maize Genetically Engineered for European Corn Borer Resistance. Phytopathology.

[37] Pleadin J., Vasilj V., Petrović D., Frece J., Vahčić N., Jahić S., Markov K. (2017). Annual Variations of Fusarium Mycotoxins in Unprocessed Maize, Wheat and Barley from Bosnia and Herzegovina. Croat. J. Food Sci. Technol..

[38] Kos J., Hajnal E.J., Šarić B., Jovanov P., Nedeljković N., Milovanović I., Krulj J. (2017). The Influence of Climate Conditions on the Occurrence of Deoxynivalenol in Maize Harvested in Serbia during 2013–2015. Food Control.

[39] Paul P.A., El-Allaf S.M., Lipps P.E., Madden L.V. (2004). Rain Splash Dispersal of *Gibberella Zeae* Within Wheat Canopies in Ohio. Phytopathology®.

[40] Doohan F.M., Brennan J., Cooke B.M., Xu X., Bailey J.A., Cooke B.M. (2003). Influence of Climatic Factors on Fusarium Species Pathogenic to Cereals. Epidemiology of Mycotoxin Producing Fungi: Under the Aegis of COST Action 835 ‘Agriculturally Important Toxigenic Fungi 1998–2003′, EU Project (QLK 1-CT-1998–01380).

[41] Blandino M., Scarpino V., Giordano D., Sulyok M., Krska R., Vanara F., Reyneri A. (2017). Impact of Sowing Time, Hybrid and Environmental Conditions on the Contamination of Maize by Emerging Mycotoxins and Fungal Metabolites. Ital. J. Agron..

[42] Logrieco A., Battilani P., Leggieri M.C., Jiang Y., Haesaert G., Lanubile A., Mahuku G., Mesterházy A., Ortega-Beltran A., Pasti M. (2021). Perspectives on Global Mycotoxin Issues and Management From the MycoKey Maize Working Group. Plant Dis..

[43] Boutigny A.-L., Richard-Forget F., Barreau C. (2008). Natural Mechanisms for Cereal Resistance to the Accumulation of Fusarium Trichothecenes. Eur. J. Plant Pathol..

[44] Leslie J.F., Logrieco A.F. (2014). Mycotoxin Reduction in Grain Chains.

[45] Degraeve S., Madege R.R., Audenaert K., Kamala A., Ortiz J., Kimanya M., Tiisekwa B., De Meulenaer B., Haesaert G. (2016). Impact of Local Pre-Harvest Management Practices in Maize on the Occurrence of Fusarium Species and Associated Mycotoxins in Two Agro-Ecosystems in Tanzania. Food Control.

[46] Miller S.S., Reid L.M., Harris L.J. (2007). Colonization of Maize Silks by *Fusarium graminearum*, the Causative Organism of Gibberella Ear Rot. Can. J. Bot..

[47] Marín P., Magan N., Vázquez C., González-Jaén M.T. (2010). Differential Effect of Environmental Conditions on the Growth and Regulation of the Fumonisin Biosynthetic Gene FUM1 in the Maize Pathogens and Fumonisin Producers Fusarium Verticillioides and Fusarium Proliferatum: Ecophysiology of F. Verticillioides and F. Proliferatum. FEMS Microbiol. Ecol..

[48] Pfordt A., Romero L.R., Schiwek S., Karlovsky P., von Tiedemann A. (2020). Impact of Environmental Conditions and Agronomic Practices on the Prevalence of Fusarium Species Associated with Ear- and Stalk Rot in Maize. Pathogens.

[49] Picot A., Hourcade-Marcolla D., Barreau C., Pinson-Gadais L., Caron D., Richard-Forget F., Lannou C. (2012). Interactions between Fusarium Verticillioides and *Fusarium graminearum* in Maize Ears and Consequences for Fungal Development and Mycotoxin Accumulation: Fusarium Spp. Interactions in Maize Ears. Plant Pathol..

[50] Fels-klerx H.J., van der Booij C.J.H. (2010). Perspectives for geographically oriented management of fusarium mycotoxins in the cereal supply chain. J. Food Prot..

[51] Van Asselt E.D., Booij C.J.H., Fels-Klerx H.J. (2012). van der Modelling Mycotoxin Formation by *Fusarium graminearum* in Maize in The Netherlands. Food Addit. Contam. Part A.

[52] Hooker D.C., Schaafsma A.W. (2005). Agronomic and Environmental Impacts on Concentrations of Deoxynivalenol and Fumonisin B1 in Corn across Ontario. Can. J. Plant Pathol..

[53] Roucou A., Bergez C., Méléard B., Orlando B. (2021). A Fumonisin Prevention Tool for Targeting and Ranking Agroclimatic Conditions Favoring Exposure in French Maize-Growing Areas. Toxins.

[54] Wu F., Bhatnagar D., Bui-Klimke T., Carbone I., Hellmich R., Munkvold G., Paul P., Payne G., Takle E. (2011). Climate Change Impacts on Mycotoxin Risks in US Maize. World Mycotoxin J..

[55] Jajić I., Jurić V., Glamočić D., Abramović B. (2008). Occurrence of Deoxynivalenol in Maize and Wheat in Serbia. Int. J. Mol. Sci..

[56] Giorni P., Bertuzzi T., Battilani P. (2019). Impact of Fungi Co-Occurrence on Mycotoxin Contamination in Maize During the Growing Season. Front. Microbiol..

[57] Vandicke J., De Visschere K., Croubels S., De Saeger S., Audenaert K., Haesaert G. (2019). Mycotoxins in Flanders’ Fields: Occurrence and Correlations with Fusarium Species in Whole-Plant Harvested Maize. Microorganisms.

[58] Deudon O. Interest and implementation of a spatialization method of meteorological data used in agricultural decision support tools. Proceedings of the 2017 EFFITA WCCA Congress.

[59] Hastie T., Taylor J., Tibshirani R., Walther G. (2007). Forward Stagewise Regression and the Monotone Lasso. Electron. J. Stat..

[60] Anbari M.E. (2011). Regularization and variable selection using penalized lieklihood. Ph.D. Thesis.

[61] Genuer R., Poggi J.-M., Tuleau-Malot C. (2010). Variable selection using random forests. Pattern Recognit. Lett..

[62] Fox J., Weisberg S. (2011). Multivariate Linear Models in R. An R Companion to Applied Regression.

[63] Hothorn T., Bretz F., Westfall P. (2020). Simultaneous Inference in General Parametric Models.

[64] Lenth R.V. (2017). Using Lsmeans.

[65] R Development Core Team (2020). R: A Language and Environment for Statistical Computing.

